# Interplay between vacancy-induced hydrogen segregation and stress-induced vacancy redistribution causing embrittlement of alpha-iron

**DOI:** 10.1080/14686996.2025.2459060

**Published:** 2025-02-03

**Authors:** Mugilgeethan Vijendran, Ryosuke Matsumoto

**Affiliations:** a Department of Mechanical and Electrical Systems Engineering, Kyoto University of Advanced Science, Kyoto, Japan; b Faculty of Engineering, University of Jaffna, Kilinochchi, Sri Lanka

**Keywords:** Intergranular failure, Iron, tensile behavior, hydrogen, vacancy-induced

## Abstract

This study proposes a novel mechanism of intergranular fracture in alpha-iron, focusing on the effects of trapped vacancies, H atoms, and their synergistic interplay under tensile strain. We present a methodology for the introduction of H into grain boundaries (GBs) resulting in a realistic distribution by considering H–H interactions. Accordingly, optimal H concentrations were determined under specific environmental conditions for GBs with and without vacancy-induced segregation under zero and 2% tensile strain, respectively. Subsequently, the reduction in cohesive energy at GBs was evaluated at the optimal H concentration under these conditions. In the case of H segregation without vacancies at zero applied strain, the reduction in the cohesive energy ranged approximately from 15% to 35% for all the GB configurations. Eventually, vacancy segregation increased H concentration at the GBs, defined as vacancy-induced H segregation. The vacancy-induced H segregation resulted in a 60–117% increase in H concentration and a 70–80% decrease in cohesive energy at a vacancy concentration of $7.49\;1/nm^{2}$ under zero applied strain. The proposed vacancy-induced H-segregation mechanism explained the delayed fracture in steel. Furthermore, the effect of tensile strain on embrittlement was elucidated, with strain-induced vacancy redistribution and vacancy-induced H segregation synergistically promoting GB decohesion, resulting in a 73–93% reduction in cohesive energy at the same vacancy concentration.

## IMPACT STATEMENT

This study introduces a novel mechanism to elucidate delayed fracture in bcc iron, focusing on the synergistic interplay between vacancies and hydrogen at symmetrical tilted grain boundaries.

## GRAPHICAL ABSTRACT

**Figure:**
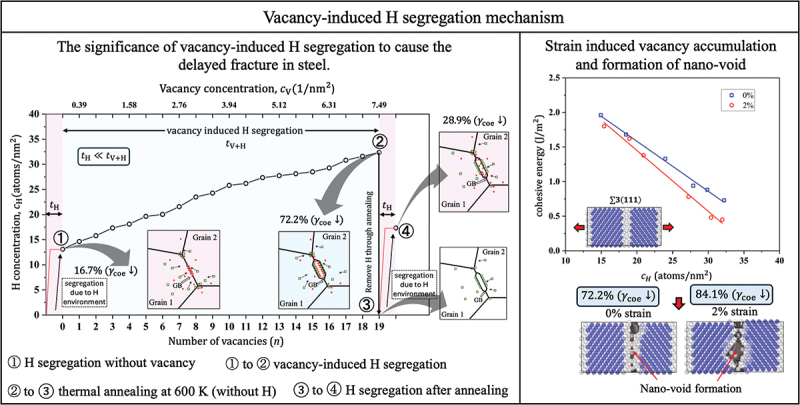

## 1. Introduction

Hydrogen is widely recognized as a sustainable energy carrier because of its exceptional energy density per unit mass [1,2]. However, ensuring its safe and efficient storage and transportation is challenging [3]. Low-alloy steel is a promising material for hydrogen storage and transportation owing to its optimal balance between cost and strength. Nevertheless, the persistent issue of hydrogen embrittlement (HE) in steels remains a considerable challenge, causing delayed fracturing on high-strength steels [4,5]. The atomistic mechanisms governing HE in this context are yet to be comprehensively elucidated, highlighting an essential area for further investigation [6]. The absence of impurities in α-iron makes it an ideal model for HE studies, as impurities can complicate the interpretation of empirical results and numerical simulations. Extensive understanding of the interactions of H with defects across diverse H concentrations under specific loading and environmental conditions is indispensable for delineating the fundamental mechanisms of HE and exploring their synergistic interplay. Studies have suggested that dislocations, stacking faults, and atomic vacancies are potential factors controlling HE [7]. Numerous theories have been proposed in this regard, including hydrogen-enhanced decohesion (HEDE) [8], hydrogen-enhanced localized plasticity (HELP) [9], and hydrogen-enhanced strain-induced vacancy (HESIV) [10]. The HEDE mechanism suggests that dissolved H at a grain boundary (GB) or crack tip weakens interatomic bonds, thereby reducing fracture energy. This explanation of embrittlement is widely utilized to elucidate the H-induced ductile-to-brittle transition [11,12]. However, the HEDE mechanism does not directly address the H-induced increase in plasticity, which is a crucial aspect of the HELP mechanism supported by various experiments [13,14]. However, atomistic studies have demonstrated that H could impede the movement of edge dislocations [15,16]. Moreover, the interaction of H with GBs and vacancies can also affect plasticity [17]. Hence, the elucidation of the interactions between GB, vacancies, and H is essential for understanding HE.

Hydrogen facilitates the formation of monovacancies and small vacancy clusters, such as di- and trivacancies, eventually leading to the formation of vacancy–hydrogen complexes. During aging without H, monovacancies dissipate or coalesce into planar or spherical vacancy clusters [18], which can nucleate as prismatic dislocation loops (PDLs) [19,20]. Experimental and simulation studies have reported the existence of PDLs with a 1/2⟨111⟩ Burgers vector in body-centered cubic (bcc) Fe [19–24]. A recent study established the viability of high-speed vacancy transport facilitated by the rapid thermally activated one-dimensional (1D) diffusion of PDLs in bcc Fe. Importantly, diffusion is hindered in regions subjected to tensile stress, implying the accumulation of PDLs near stress singularities [25]. Consequently, substantial vacancy accumulation in the tensile stress region could result from the transition between PDL and vacancy clusters and the effect of stress on this transition [19]. Nevertheless, the direct relationship between the accumulation of vacancies and the resulting fracture remains unclear [26].

High-strength steel is notably susceptible to significant embrittlement because of H-induced intergranular (IG) fracture [27–30]. Comprehensive atomistic studies have demonstrated that the presence of H atoms can reduce the cohesive energy of GBs [31–36]. However, the optimum H concentration required to induce such a fracture and the mechanism that can trigger this concentration are yet to be determined [37]. Moreover, extensive experimental and atomistic simulation studies have revealed that H-related IG fracture involves not only H-induced decohesion but also the generation of vacancies due to localized plastic deformation near GBs [26,38,39]. The evidence of void formation and coalescence through crack growth along GBs has been reported in a recent experimental study [40]. Furthermore, first-principle calculations have confirmed the effects of vacancies at Σ3-type tilt GBs on HE directed by the V–H complex formed during fracture [4]. A recent study investigated the impact of vacancies at GBs, particularly those with higher carbon content, revealing a considerable reduction in cohesive energy. This finding aligns with experimental results and suggests that vacancies, along with H and carbon, promote the occurrence of delayed fracture [41]. Delayed fracture caused by H is characterized by sudden fracture occurring after prolonged exposure to stress and an H-rich environment. Explaining delayed fracture solely based on H concentration is challenging because H diffusion is rapid, and thermal equilibrium is easily attained in bcc Fe [42]. Similarly, molecular dynamic analyses under mechanical loading confirmed the propensity of H and vacancies to form V–H clusters at Σ3-type tilt GBs. This interplay promotes the nucleation of nanovoids and premature fracture at GBs [43]. Although previous studies confirmed various mechanisms of decohesion, the mechanism of H and vacancy segregation as well as their effects on embrittlement must be comprehensively elucidated under external strain and without strain at various GBs.

The present study was conducted to clarify the interplay between vacancies and H at symmetric-tilt GBs (STGBs), ultimately resulting in embrittlement. Section 2 introduces a novel method for examining H segregation at STGBs, accounting for H–H interactions. Additionally, the vacancy-induced H-segregation mechanism is introduced, and the resulting reduction in cohesive energy is evaluated, considering the effects of mobile and immobile H at the IG fracture surface (FS). In Section 3, the effects of H segregation at different STGBs are discussed, considering their correlations with GB energy, free volume, and volume expansion at the interstitial sites. Moreover, the effect of tensile strain on the H/vacancy-induced H accumulation and resulting GB decohesion is analyzed, thus clarifying vacancy-induced H segregation in the context of delayed fracture. Furthermore, stress-induced vacancy accumulation and its localization resulting in the formation of voids are elucidated in the context of GB weakening.

## 2. Simulation model and methodology

The analyses presented in this study were performed using the Large-scale Atomic/Molecular Massively Parallel Simulator (LAMMPS), open-source software for molecular dynamics simulations [44]. The Fe–Fe, Fe–H, and H–H interactions were described through the embedded atom method potential developed by Wen [45] based on the Fe–Fe model of Ackland-2004 empirical potential [46]. The embedded atom potential can accurately represent the heat of H solution and the interaction energy between H atoms and different lattice defects in α-Fe [47,48].

### 2.1. Grain boundary construction

To obtain the $\left[110\right]$ STGBs (hereafter referred to as GBs), two crystals with intended crystallographic orientations were constructed in a rectangular simulation box and joined along the GB plane. As certain atomic pairs became excessively close during the GB plane joining, we removed one of the excessively close atoms in a pair and relocated the remaining atom to the GB plane. Table 1 presents detailed information regarding the GBs considered in this study.

**Table 1.** Detailed information on STGBs with misorientation angles and cell dimensions.

	Unit vector			Misorientation angle	Dimension (nm)		
GB-type	x	y	z	$\left({\;}^{\circ }\right)$	x	y	z
$\sum 19\left(116\right)$	$\left[1\overset{ˉ}{1}\overset{ˉ}{6}\right]$	$\left[110\right]$	$\left[3\overset{ˉ}{3}2\right]$	26.53	7.04	1.21	1.87
$\sum 9\left(114\right)$	$\left[\overset{ˉ}{1}14\right]$	$\left[110\right]$	$\left[\overset{ˉ}{2}2\overset{ˉ}{1}\right]$	38.94	4.77	1.21	1.71
$\sum 11\left(113\right)$	$\left[1\overset{ˉ}{1}\overset{ˉ}{3}\right]$	$\left[110\right]$	$\left[3\overset{ˉ}{3}2\right]$	50.48	3.67	1.21	1.34
$\sum 3\left(111\right)$	$\left[\overset{ˉ}{1}1\overset{ˉ}{1}\right]$	$\left[110\right]$	$\left[1\overset{ˉ}{1}\overset{ˉ}{2}\right]$	70.53	3.95	1.21	2.09
$\sum 17\left(223\right)$	$\left[\overset{ˉ}{2}23\right]$	$\left[110\right]$	$\left[\overset{ˉ}{3}3\overset{ˉ}{4}\right]$	86.63	4.70	1.21	1.67

In our calculations, we implemented the three-dimensional periodic boundary condition, thus obtaining two GBs with identical misorientation angles within the unit cell. The stable configuration of GBs was obtained through the relaxation of atomic configuration and simulation cell size using the conjugate gradient method. Accordingly, GB energy and free volume around the GB were evaluated. The GB energies were calculated using Eq (1):

(1) $$\gamma_{\mathrm{GB}}=\frac{E_{\mathrm{GB}}-nE_{\mathrm{Fe}}}{2A_{\mathrm{GB}}}$$

where $E_{\mathrm{GB}}$ is the energy of the system with a GB, $E_{\mathrm{Fe}}$ is the energy of one atom in the undeformed bcc structure, n is the number of Fe atoms in the GB model, and $A_{\mathrm{GB}}$ is the area of one GB in the simulation model. The free volume at the GB was evaluated as the excess volume per unit $A_{\mathrm{GB}}$ using the following equation:

(2) $$\Omega =\frac{V_{\mathrm{GB}}-nV_{\mathrm{Fe}}}{2A_{\mathrm{GB}}}$$

where $V_{\mathrm{GB}}$ is the volume of the system with a GB, $V_{\mathrm{Fe}}$ is the volume of one atom in the undeformed bcc structure, and n is the number of Fe atoms in the GB model.

### 2.2. Stable hydrogen distribution at realistic concentrations in GB models

**Figure 1.**
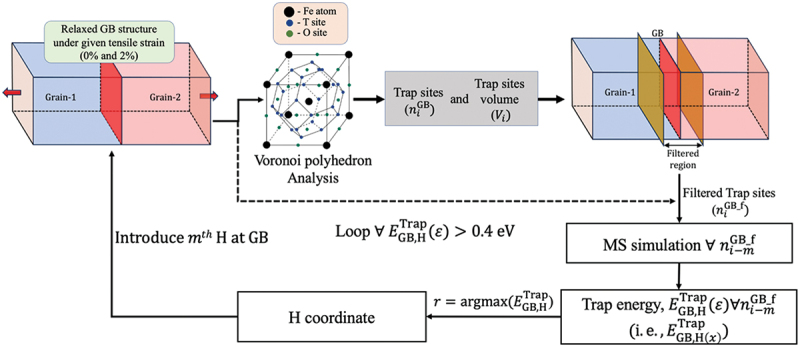
Schematic of the proposed algorithm (color).

To analyze the effect of H on HE, the distribution of H atoms at stable positions must reasonably represent real conditions. Thus, we propose a novel approach for introducing H into defects (i.e. GBs in this case) based on trap energy. The approach has several merits compared to the grand canonical Monte Carlo (GCMC) method. GCMC can provide the H distribution and the equilibrium H concentration at a given chemical potential of H. However, the evaluation of trap energy as a function of the number of H or vacancies is complicated using this method. At the same time, the proposed methodology yields the equilibrium H concentration and distribution, and defines the characteristics of H trapping at different GBs depending on the trap energy. Therefore, the proposed methodology is beneficial for elucidating the driving force of H trapping at GBs. As the potential H sites are determined by the structure and the insertion is determined by trap energy, the computational cost of the suggested strategy is lower than that of GCMC. This approach for introducing H at the GBs utilizes a Python-based in-house algorithm (Figure 1, where x denotes the coordinates of interstitial sites). In this algorithm, first, possible sites for H insertion within the GB are identified using Voronoi polyhedron analysis. In the perfect bcc lattice, tetrahedral sites (T-sites) correspond to the vertices of the Voronoi polyhedron, whereas octahedral sites (O-sites) correspond to the centers of squares on the polyhedron surfaces (Figure 1). These rules were used to estimate the sites for H insertion. Subsequently, the Voronoi polyhedron analysis was used for the positions of Fe. After the identification of potential sites, we performed the Voronoi polyhedron analysis again to estimate the volume of each site as a volume of each Voronoi polyhedron. The dashed line in Figure 1 details the progression of the algorithm after the identification of potential sites. The volume of each trap site is evaluated, and the variation in the volume near the GB is assessed. In this context, the GB free volume (Eq (2)) is approximately equal to the sum of volume expansions near the GB. Herein, volume expansion refers to the increase in the interstitial site volume near the GB relative to that in the bulk. These evaluations are discussed in Section 3.

The structure is distorted in the region near the GB. Therefore, the H atoms occupying the T-sites and the O-sites were considered. Further, the model includes two GB planes, but H was introduced only in one of them. Once the possible sites were identified, the algorithm calculated the most stable positions (those with the highest trapping energy) for the introduction of H atoms. The trap energy for each site was computed as:

(3) $$E_{GB,H\left(x\right)}^{\mathrm{Trap}}\left(\varepsilon \right)=-\left[\left\{E_{\mathrm{GB},mH}\left(\varepsilon \right)-E_{\mathrm{GB},\left(m-1\right)H}\left(\varepsilon \right)\right\}\text{\textbackslash{}break}-\left\{E_{B,H}\left(\varepsilon \right)-E_{B}\left(\varepsilon \right)\right\}\right]$$

where, $E_{\mathrm{GB},mH}\left(\varepsilon \right)$ is the potential energy of the system with GB and the $m^{\mathrm{th}}$ H atom at a trap site, $E_{\mathrm{GB},\left(m-1\right)H}\left(\varepsilon \right)$ is the potential energy of the system with GB with the ${\left(m-1\right)}^{\mathrm{th}}$H atom (m is the number of H at each loop, i.e. $m=\left\{1,2,\ldots ,m^{*}\right\}$), $E_{B,H}$ is the potential energy of the perfect crystal with an H atom, $E_{B}$ is the potential energy of the perfect crystal, and ε is the applied strain as described below.

To obtain a realistic H distribution, the effects of H–H interactions must be considered, ensuring that the potential positioning of the next H atom is contingent upon the preceding H distribution. As previously elucidated, H atoms were sequentially introduced by evaluating all potential sites and selecting that with the largest trap energy. Subsequently, after inserting the chosen H atom at the selected position, the possible positions for the subsequent H atom were assessed and selected using the same method (Figure 1). This method ensures a realistic H distribution considering H–H interactions. Similarly, the precise definition of the H concentration is pivotal, as it profoundly affects the interpretation of GB–H interactions and their subsequent behavior. Herein, we defined the concentration using the relationship between H occupancy $C_{x}$, under thermal equilibrium and the H occupancy at the T-site, $C_{T}$, in non-deformed bcc-Fe lattice [49]:

(4) $$\frac{C_{x}}{1-C_{x}}=\frac{C_{T}}{1-C_{T}}\exp\left(\frac{E_{GB,H\left(x\right)}^{T\mathrm{rap}}}{k_{B}T}\right)$$

where $k_{B}$ and T are Boltzmann constant and temperature, respectively.

The $C_{T}=0.9686\times 10^{-6}\sqrt{p}\exp\left(-3440/T\right)$ expresses $C_{T}$ as an empirical function of the environmental pressure p and temperature T [32,42,50]. The calculations were performed for 70 MPa and 300 K (i.e. conditions in pipes and tanks of fuel cell vehicles). Under these conditions, the concentration is defined by the stable occupancy for $E_{GB,H\left(x\right)}^{\mathrm{Trap}}$ >0.4 eV, where $C_{x}$ rapidly increases, reaching $C_{x}\approx 0.5$ for $E_{GB,H\left(x\right)}^{\mathrm{Trap}}\approx$ 0.4 eV in the high-pressure hydrogen environment. Certainly, at finite temperatures, some H atoms can be de-trapped from sites with energies above 0.4 eV, while others remain trapped at sites with energy below 0.4 eV. Additionally, the trapping order can vary, but we assumed that the total number of H and the trap energy profile do not considerably change in this evaluation procedure.

The following two parameters: $m^{*}$ and $C_{H}$ respectively denote the equilibrium number of H atoms at the GB and the H concentration representing the $m^{*}$ per unit $A_{\mathrm{GB}}$. The calculations were performed without applied external strain and under a tensile strain of 2%, with the strain direction $\left(x\right)$ normal to the GB plane. Similarly, for both cases (with and without strain), a two-stage analysis was conducted. Initially, H segregation and concentration were defined without considering vacancies, as explained above. Subsequently, an analysis of vacancy-induced H segregation was conducted to define the new stable $C_{H}$ at GBs. The detailed methodology is elucidated in Section 2.3. The energies were obtained through the relaxation of atomic configuration and simulation cell using the conjugation gradient method along the *y* or *z* direction.

The generalized McLean models with the extension to multiple segregation sites at the GB [51], indeed provide a more robust framework for addressing multi-component segregation with multiple sites. However, we adopted a single-site approach, identifying the strongest trapping site for H insertion rather considering multiple strong sites. Because, the challenge arises in defining subsequent H positions, as the strength of some sites may weaken as others are occupied. This makes it difficult to achieve equilibrium states computationally, as such calculations are extremely expensive. Equilibrium concentrations depend on the trapping energy, and our model incorporates both H–H interactions and H–V (see Section 2.3) interactions. This allows us to address the discrepancies introduced by the simplified models we used. Additionally, it is important to note that we iteratively introduced vacancies after H atoms, rather than simultaneously. This approach reflects the ability of H to quickly reach an equilibrium state.

### 2.3. Evaluation of vacancy-induced hydrogen segregation at GBs

To investigate the impact of H, vacancies, and their synergetic interplay on the cohesive energy of GBs, we further addressed the impact of vacancy trapping in stabilizing H at the GBs. Once the optimal $C_{H}$ at the GBs was determined, a vacancy was introduced at the most stable position (that with the highest vacancy trap energy). Iteratively, Fe atoms were removed one by one, their trap energy was evaluated using the following equation:

(5) $$E_{Vn}^{\mathrm{Trap}}\left(\varepsilon \right)=-\left[\left\{E_{\mathrm{GB},m^{*}H,Vi}\left(\varepsilon \right)-E_{B,m^{*}H,V\left(i-1\right)}\left(\varepsilon \right)\right\}\text{\textbackslash{}break}-\left\{E_{B,V}\left(\varepsilon \right)-E_{B}\left(\varepsilon \right)\right\}\right]$$

where, $E_{\mathrm{GB},m^{*}H,Vi}\left(\varepsilon \right)$ is the total energy of GB-H (the GB with H) with $i^{\mathrm{th}}$vacancy (i.e. $i=\left\{1,2,\ldots ,n\right\}$), $E_{B,V}\left(\varepsilon \right)$ is the total energy of the perfect crystal with a vacancy, and $E_{B}\left(\varepsilon \right)$ is the total energy of the perfect crystal.

Once a vacancy was introduced, a similar analysis was conducted to evaluate the nature of the H trapping, as explained in Section 2.2. The corresponding change in $C_{H}$ was assessed for each introduced vacancy. This process continued until the vacancy concentration ($C_{V})$ reached 7.49 1/nm^2^, resulting in a reduction in the cohesive energy of a GB by almost 70–80%, as discussed in Section 3. As many vacancies are introduced, the H site and trap energy might change slightly. Nevertheless, the energy gain by H trapping will not change, thus the calculation of cohesive energy reduction will not change considerably. Further details, including the entropy of defects and its impact on these calculations, are provided in the Appendix A.

### 2.4. Evaluation of the work of separation (cohesive energy) of GBs

**Figure 2.**
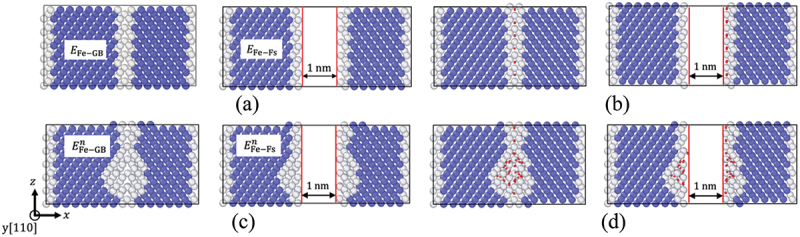
Models used to calculate GBs and free surface energy at the GB: (a) without H and vacancies, (b) with initial H segregation but without vacancies, (c) with vacancies, and (d) with vacancies and H segregation. Blue, white, and red circles denote bcc Fe, non-bcc Fe, and H atoms, respectively (color).

We calculated the cohesive energy of GBs at four stages: (i) before introducing H and vacancies, (ii) after H segregation without vacancies, (iii) after the introduction of vacancies (i.e. affected only by vacancies), and (iv) after vacancy-induced H segregation. Figure 2(a,d) illustrates the four calculation stages. In the first stage, the cohesive energy is determined by the baseline cohesive energy of the system without H and vacancies.

The cohesive energy ($\gamma_{\mathrm{coe}})$ was evaluated using the following equation:

(6) $$\gamma_{\mathrm{coe}}=\frac{E_{\mathrm{Fe}-\mathrm{Fs}}-E_{\mathrm{Fe}-\mathrm{GB}}}{A_{\mathrm{GB}}}$$

where $E_{\mathrm{Fe}-\mathrm{Fs}}$ is the energy of the model (with or without vacancies/H) with free surfaces formed at a GB plane, and $E_{\mathrm{Fe}-\mathrm{GB}}$ is the energy of the system with a GB (Figure 2(a)).

In stage (iii), cohesive energy $\gamma_{coe}^{n}$ (n denotes the number of vacancies) was evaluated as in stage (i), using Eq (6). The model was constructed with a consistent number of vacancies, and their positions were maintained during separation, as discussed below. In cases (ii) and (iv), the impact of H segregation on the cohesive energy was evaluated considering no H diffusion to/along the GB during the fracture.

However, the effect of H mobility during fracture was also investigated for the selected GB model, as discussed in Section 3.3. Hydrogen atoms tend to segregate very close to the GB plane because of the presence of stable trap sites there. Consequently, several H atoms appear on the surfaces when the material is separated at the GB plane. Accordingly, the cohesive energy was assessed by estimating the trapping energy of H atoms to the surface through the following equation:

(7) $$\gamma_{coe}^{m,n}=\gamma_{coe}^{n}-\underset{i}{\sum }\frac{\left(E_{FS,H\left(x\right)}^{T\mathrm{rap}}-E_{GB,H\left(x\right)}^{T\mathrm{rap}}\right)}{A_{\mathrm{GB}}}$$

where $E_{FS,H\left(x\right)}^{\mathrm{Trap}}$ is the H trapping energy on the free surface (GB planes), and $E_{GB,H\left(x\right)}^{\mathrm{Trap}}$ is the trapping energy of each H atom at the GB. In Eq (7), the notations differ before the introduction of vacancies (i.e. case (ii)). For instance, $\gamma_{coe}^{m,n}$ is replaced by $\gamma_{coe}^{m}$, and $\gamma_{coe}^{n}$ is replaced by $\gamma_{\mathrm{coe}}$, where $\gamma_{coe}^{m,n}$ indicates the cohesive energy of a GB with m H atoms and n vacancies.

**Figure 3.**
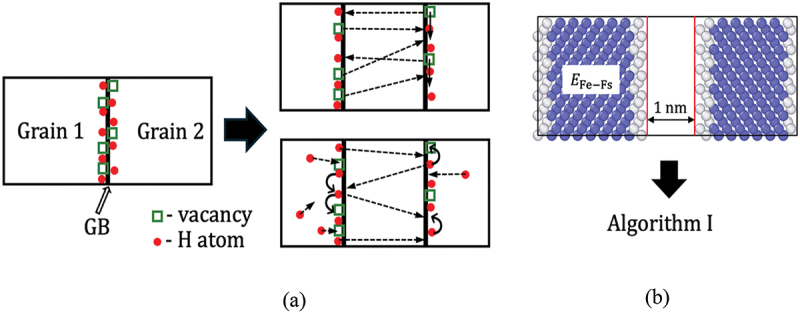
Schematic of (a) H and vacancy diffusion during fracture, (b) model used to analyze H trapping at FS. The dashed arrows indicate the motion of H or vacancies across the GB/FS and H diffusion from the bulk to the GB/FS during fracture, and solid arrows indicate diffusion of H and vacancies along the GB/FS during fracture (color).

Considering these calculations, we further examined the reduction in cohesive energy due to mobile H, which can diffuse on the gradually formed FSs. However, considering the computational costs, we defined such behavior only for ∑3 STGB. In this context, it is crucial to consider two main behaviors. First, during separation, H can diffuse to more stable positions; second, during fracture, $C_{H}$ at FSs tends to increase owing to the high diffusivity of H in bulk iron (Figure 3(a)).

Accordingly, after the introduction of the free surface at the GB plane, as shown in Figure 3(b), a similar analysis was conducted as outlined in Section 2.2 to compare the distributions and concentrations of H. Yamaguchi et al. proposed an equation to quantify the IG cohesive energy caused by mobile and immobile H related to different $C_{H}$ at an FS [52]:

$$\gamma_{coe}^{m}=\gamma_{\mathrm{coe}}-\sum_{i}E_{GB,H\left(x\right)}^{\text{Trap}}A_{\mathrm{GB}}+\sum i\frac{E_{FS,H\left(x\right)}^{\text{Trap}}}{A_{\mathrm{GB}}}$$

(8) $$\;-\frac{T\left[2S_{c}\left(\Gamma_{i}\right)N_{\mathrm{FS}}-S_{c}\left(\Gamma_{\mathrm{GB}}\right)N_{\mathrm{GB}}\right]}{A_{\mathrm{GB}}}-\frac{\left(2n_{i}-n_{\mathrm{GB}}\right)\mu_{b}}{A_{\mathrm{GB}}}$$

where Γ is the ratio of segregated/trapped H atoms ($n_{\mathrm{GB}/\mathrm{FS}})$ to possible trap sites per $A_{\mathrm{GB}}$ ($N_{\mathrm{GB}/\mathrm{FS}})$ at 0 K, $S_{c}$ is the configurational entropy per single atom given by $-k_{B}\left[\Gamma \ln\Gamma +\;\left(1-\Gamma \right)\ln\left(1-\Gamma \right)\right]$, $\mu_{b}$ is the chemical potential in the lattice given by $k_{B}T\ln\left[\Gamma_{B}/\left(1-\Gamma_{B}\right)\right],$ where $\Gamma_{B}$ is lattice bulk H concentration. $\Gamma_{i}$ and $n_{i}$ can be expressed as $n_{\mathrm{GB}}/(2N_{\mathrm{FS}}$) and $n_{\mathrm{GB}}/2$ assuming constant composition (i.e. fast fracture), and $\Gamma_{\mathrm{FS}}$, $n_{\mathrm{FS}}$ assuming constant chemical potential (i.e. slow fracture), respectively. Subscripts GB, FS, and B represent the GB, FS, and bulk, respectively. For the case of GB/FS with vacancies, $\gamma_{coe}^{m}$ is replaced by $\gamma_{coe}^{m,n}$, and $\gamma_{\mathrm{coe}}$ is replaced by $\gamma_{coe}^{n}$.

Similarly, the vacancy diffusion during fracture exhibits similar characteristics to H diffusion. However, the diffusion barrier for the vacancies in the [110] STGB is larger than that for H diffusion. For instance, in ∑3 GB, the migration barrier is approximately 0.95 eV [53]. Therefore, we assumed that vacancy diffusion occurs during the incubation time of delayed fracture, whereas it does not occur during fracture.

## 3. Results and discussion

**Figure 4.**
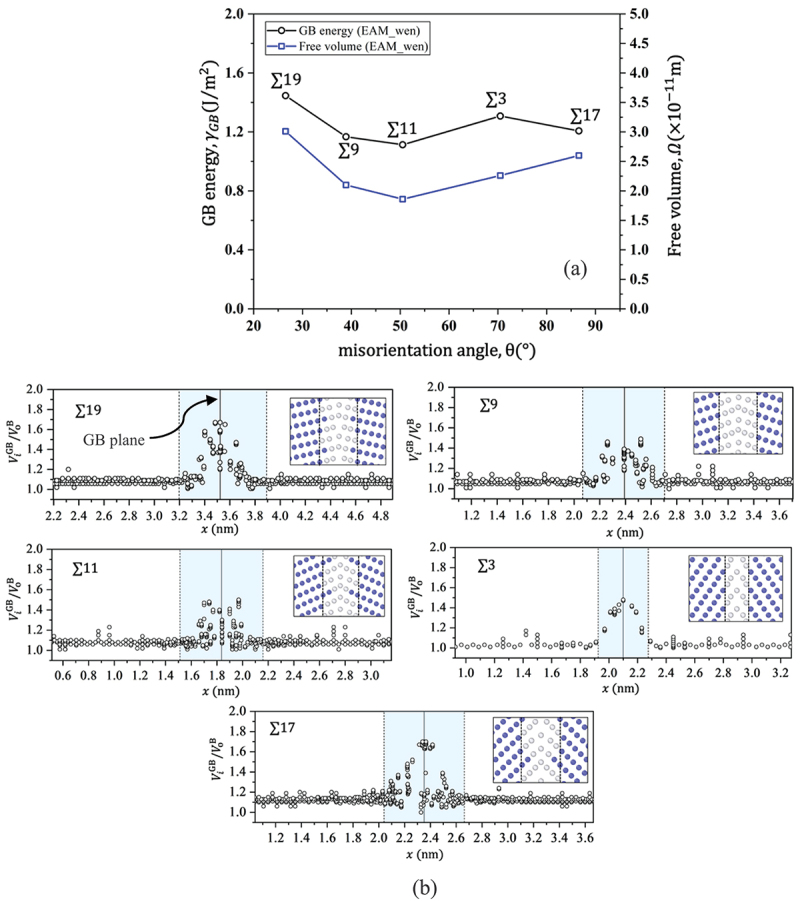
(a) Correlation between GB energy and GB free volume, (b) volume expansion of H trap sites at GBs, where shaded area indicates the range of $V_{i}^{GB}/V_{i}^{B}$ at a GB. Blue and white circles denote bcc and non-bcc Fe atoms, respectively (color).

Figure 4(a) shows the calculated GB energy and free volume as functions of the misorientation angle, revealing a strong correlation between the GB energy and free volume, except for ∑17. As explained above, free volume is the excess volume available at the GB, which can also be represented as the sum of the volumes of the H trap sites (i.e. *T*- and O-sites). Accordingly, we defined a parameter called volume expansion ($i.e.,V_{i}^{GB}/V_{o}^{B})$, which represents the volume ratio of sites at GB ($V_{i}^{GB}$) to minimum volume of O sites in the bulk ($V_{o}^{B}$) (Figure 4(b)). A strong correlation between interstitial site volume and H trapping energy has been previously reported, indicating that a larger volume corresponds to a stronger trap site [42]. Consequently, $V_{i}^{GB}/V_{o}^{B}$ characterizes the available excess volume and nature of H segregation at GBs. In Figure 4(b), a shaded region represents the volume expansion of interstitial sites at GBs. The horizontal range illustrates the distribution of excess volume along the x-direction (i.e. emphasizing excess volume near the GB plane). Additionally, a snapshot of the GB structure is given in Figure 4(b). Figure 4(a) confirms that ∑19 and ∑17 have very high free volume.

### 3.1. Hydrogen segregation at GBs without vacancies

**Figure 5.**
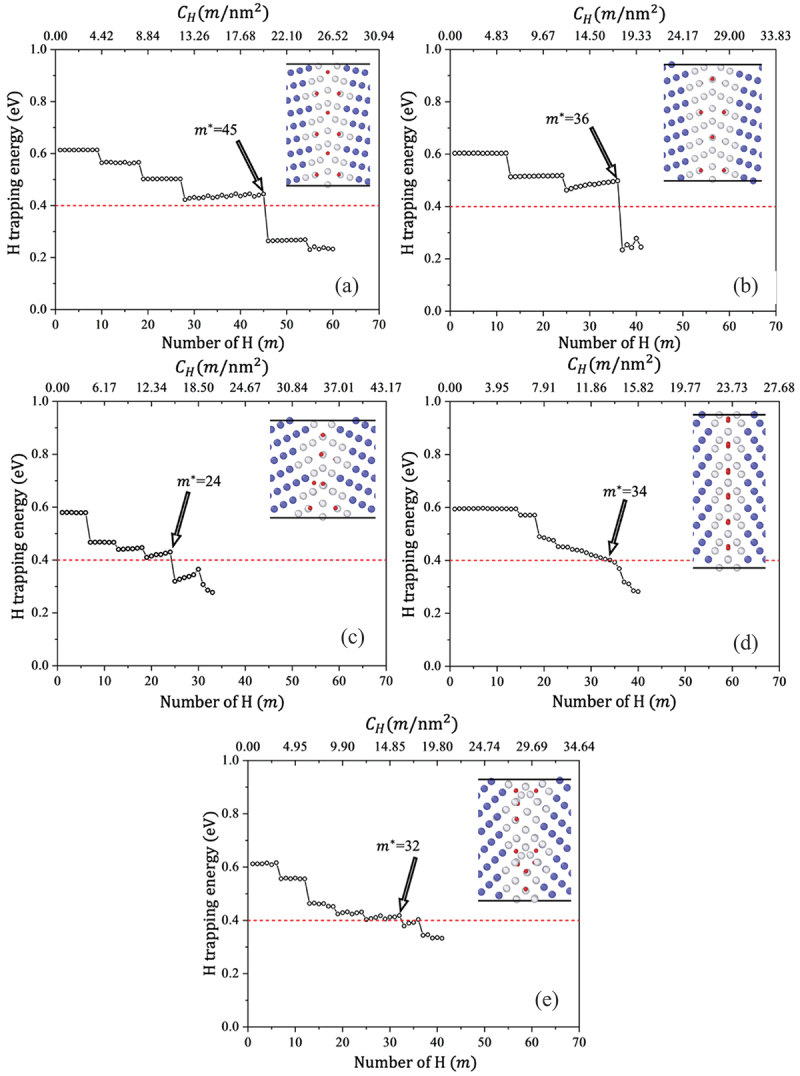
Hydrogen trapping energy vs. The number of introduced H atoms, and determination of the optimal H concentration for given environmental conditions: (a) $\sum 19\left(116\right)$, (b) $\sum 9\left(114\right)$, (c) $\sum 11\left(113\right)$, (d) $\sum 3\left(111\right)$, and (e) $\sum 17\left(223\right)$. Blue, white, and red circles denote bcc Fe, non-bcc Fe, and H atoms, respectively (color).

Realistic $C_{H}$ is vital for the comprehensive analysis of the effects of H on GBs, particularly their role in embrittlement. As explained in Section 2.2, we defined H distribution by choosing the energetically most stable configuration for each H atom, taking into account previously trapped H. Figure 5 illustrates the trapping energy as a function of the number of H (m) atoms at the GBs. The trap energy of H exhibits a step-like behavior, wherein the step height varies between GBs. Initially, H tends to trap at stronger sites (∼0.6 eV). However, as the $C_{H}$ increases, the repulsive force and the lower available free volume lead to a decrease in trapping energy. The non-monotonous trend of the H trapping energy is associated with the availability of stronger trap sites at the GB and adjacent planes (i.e. shaded area of $V_{i}^{GB}/V_{o}^{B}$; Figure 4(b)). For instance, in ∑19, ∑9, ∑11, and ∑17, H is trapped at both GB and adjacent planes, whereas in ∑3, H is only trapped at the GB plane (Figure 5). This means that in the case of ∑3, the available sites with higher free volume are localized on the GB plane (∑3 in Figure 4(b)).

#### 3.1.1. Hydrogen concentration and decohesion of GBs at zero strain

**Figure 6.**
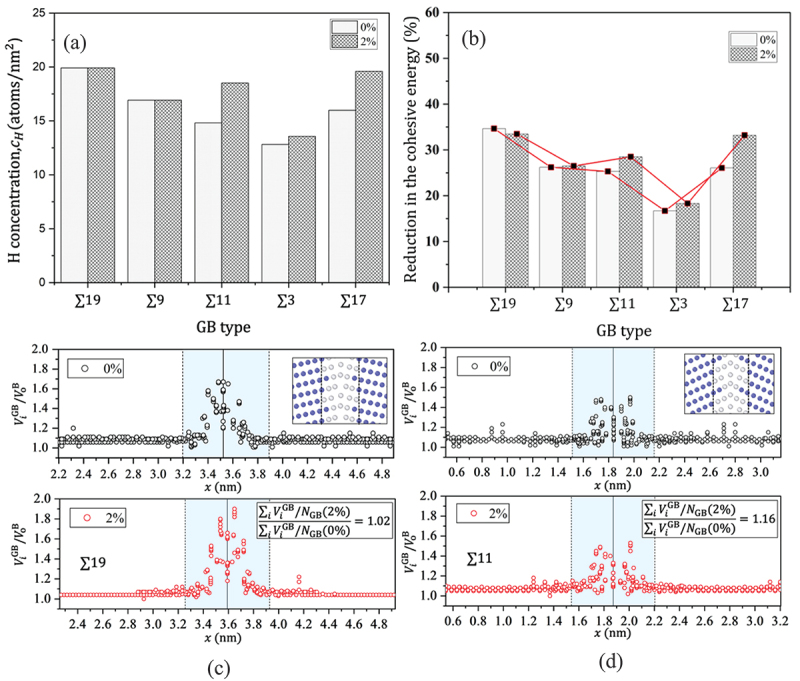
(a) H concentration for different tensile strains, (b) reduction of cohesive energy for different tensile strains, (c) and (d) volume expansion under tensile strain for ∑19 and ∑11, respectively (color).

The variation in $C_{H}$ across GBs (Figure 6(a)) is strongly correlated with the GB energy and GB-free volume (Figure 4(a,b)), except for ∑3. A first-principles calculation also revealed a strong correlation between GB structure, free volume, and $C_{H}$ [32]. The weak correlation observed in ∑3 was attributed to the substantial impact of free volume at the GB and adjacent planes. As discussed above, the larger magnitude of $V_{i}^{GB}/V_{o}^{B}$ in ∑3 is limited to the GB plane (Figure 4(b)). Therefore, H tends to be trapped at the GB plane only with stronger sites, thus reaching optimal $C_{H}$ values for further weakening the H trap site energy, while the contribution of the H–H repulsive force starts to prevail. Figure 6(b) shows the calculated reduction in cohesive energy of GBs (the red line indicates the trend of the peak value (i.e. maximum reduction in cohesive energy) at each GB), demonstrating a good correlation between a decrease in cohesive energy and $C_{H}$ (Figure 6(a)). The percentage decrease in cohesive energy was defined as ($1-\gamma_{coe}^{m}/\gamma_{\mathrm{coe}})\times 100$. Accordingly, for the optimal $C_{H}$, the decrease in cohesive energy at GBs is within only 15–35%.

#### 3.1.2. Hydrogen concentration and decohesion of GBs at 2% strain

Understanding the behavior of H segregation under tensile loading is pivotal for a comprehensive elucidation of the strain/stress process associated with HE. Figure 6(a) exhibits the effect of external tensile loading on the nature of H trapping at GBs. Notably, ∑19 and ∑9 show no sensitivity to external tensile strain, whereas in the rest of the cases, $C_{H}$ increases. We thus concluded that GBs with low misorientation angles (Figure 4(a)) are effectively unaffected by external strain, whereas high misorientation-angle GBs are sensitive to tensile strain. No sensitivity of ∑19 and ∑9 to strain was attributed to the low volume expansion under tensile strain. As discussed above, H trapping and free volume are strongly correlated. Accordingly, the change in volume expansion under tensile strain was attributed to the changes in $C_{H}$. The change in the volume expansion was evaluated as the ratio of $\underset{i}{\sum }V_{i}^{GB}/N_{\mathrm{GB}}$ at 2% to that at 0% strain. Here, $\underset{i}{\sum }V_{i}^{GB}/N_{\mathrm{GB}}$ is the average volume of the interstitial sites at the GB calculated in the shaded region in Figure 6(c) and (d). For ∑19 and ∑9, $\frac{\sum_{i}V_{i}^{GB}/N_{\mathrm{GB}}\left(2\%\right)}{\sum_{i}V_{i}^{GB}/N_{\mathrm{GB}}\left(0\%\right)}\approx 1.02$, implying negligible volume expansion caused by the tensile strain. Therefore, $C_{H}$ remains the same in ∑19 and ∑9, irrespective of external tensile strain. For ∑11, $\frac{\sum_{i}V_{i}^{GB}/N_{\mathrm{GB}}\left(2\%\right)}{\sum_{i}V_{i}^{GB}/N_{\mathrm{GB}}\left(0\%\right)}=1.16$, implying considerable volume expansion under tensile strain. Therefore, more H can be trapped at the GB and the adjacent plane. A similar tendency is observed for ∑3 and ∑17, with $C_{H}$ increasing by 1.08 and 1.19 times, respectively. ∑3 is considered a unique GB, less sensitive to trap H with and without external strain, as the trap sites are concentrated near the GB. We obtained that for the given $C_{H}$ at 2% strain, the reduction of cohesive energy was only about 20–35% in GBs. Notably, GBs with larger misorientation angles, such as ∑11 and ∑17, are sensitive to strain, with cohesive energy decreasing by approximately 3.2% and 7%, respectively. Given the relatively minor reduction in cohesive energy, the delayed fracture or strain-induced HE effect in Fe cannot be explained by H segregation without considering vacancies. In other words, a significantly higher $C_{H}$ is necessary to induce brittle fracture; therefore, the mechanisms that drive the attainment of such concentration must be investigated. Moreover, from the perspective of H diffusion time, explaining the delayed fracture that occurs after several years is also challenging.

### 3.2. Vacancy-induced hydrogen segregation at GBs

After saturating the GBs with H atoms, the effects of vacancies and their role in triggering H trapping in GBs were investigated. After the saturation of GBs with H atoms, vacancies were sequentially introduced at the most stable sites, considering their trap energies (i.e. $E_{Vn}^{\mathrm{Trap}}\left(\varepsilon \right)>1\;\mathrm{eV})$, which resulted in an increase in $C_{H}$. As explained above, this process was continued until a $C_{V}$ of 7.49 1/nm^2^ was reached. The probability for vacancy trapping was estimated by the ratio $\frac{C_{o}}{1-C_{o}}=\frac{C_{\mathrm{Vo}}}{1-C_{\mathrm{Vo}}}\exp\left(\frac{E_{Vn}^{T\mathrm{rap}}}{k_{B}T}\right)$, where $C_{o}$ is site occupancy, and $C_{\mathrm{Vo}}$ is the lattice vacancy concentration. Furthermore, assuming $C_{\mathrm{Vo}}=10^{-6}$, vacancies easily trap at GBs (i.e. $C_{o}\approx 1$). However, the $C_{\mathrm{Vo}}$ is not explicitly defined. Yet, vacancies were trapped at sites with an energy of 1 eV even at vacancy concentration of 1.5$\;\times \;10^{-17}$ for 0.5 occupancy. This implies that if a vacancy generated inside a grain diffuses to a GB, it will undoubtedly be trapped by a trap site.

#### 3.2.1. Vacancy-induced hydrogen segregation and decohesion of GBs at zero strain

**Figure 7.**
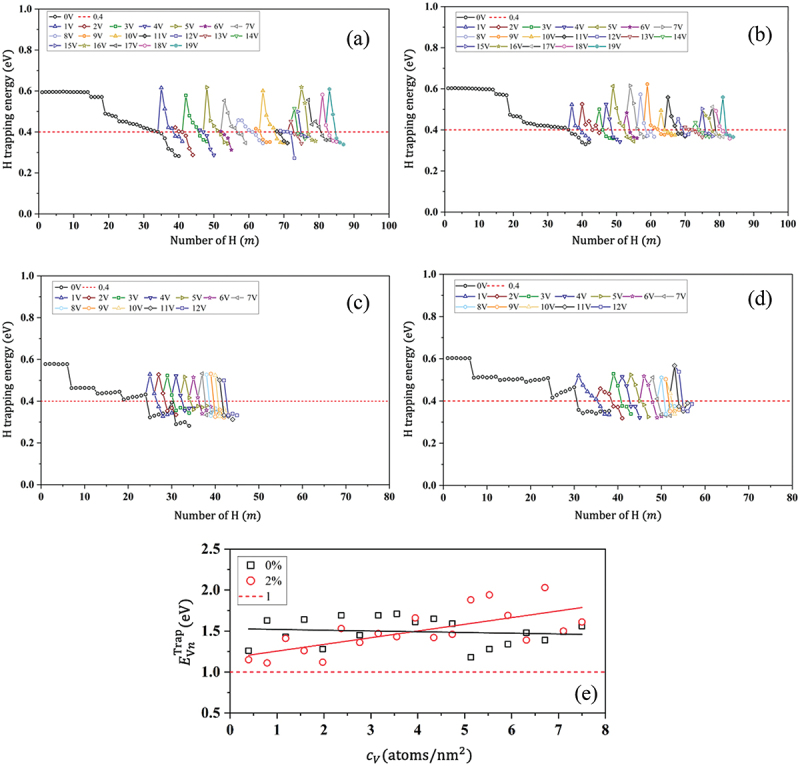
Vacancy-induced H segregation at GBs. (a), (b) $\sum 3\left(111\right)$ for 0 and 2% tensile strain, respectively; (c), (d) $\sum 11\left(113\right)$ for 0 and 2% tensile strain, respectively; and (e) vacancy trapping energy against vacancy concentration under 0 and 2% tensile strain in $\sum 3\left(111\right)$ (color).

**Figure 8.**
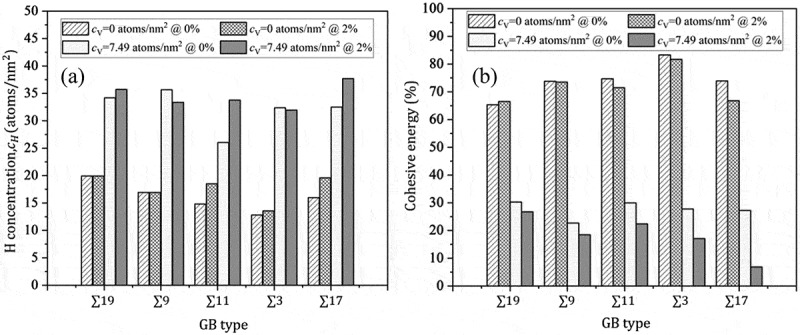
(a) The saturated H concentration with and without vacancy-induced H segregation at no strain and 2% tensile strain; (b) resulting reduction in the cohesive energy ($(\gamma_{coe}^{m}/\gamma_{\mathrm{coe}})\times 100$).

Figure 7(a–d) shows the trend of H trapping energy after the introduction of a vacancy at the GB. This trend aligns with the nature of H trapping at free volume, as H tends to occupy sites with larger free volumes. Figure 7(e) plots vacancy trap energy against vacancy concentration with and without tensile strain, as further elucidated in Section 3.2.2. For each vacancy, one to five H atoms tend to be trapped, depending on the vacancy distribution at the GB. Consequently, $C_{H}$ increases by 70–150% across all GBs (Figure 8(a)) compared to vacancy-free H segregation under zero applied strain. Figure 8(b) reveals that vacancy-induced H segregation is more prominent, where cohesive energy at GBs notably decreases. The sensitivity to vacancy-induced decohesion is observed almost for all GBs, reaching similar saturation levels in cohesive energy decrease. The reduction in the cohesive energy is greater by 100–330% (Figure 8(b)) than the vacancy-free H segregation without applied strain. Therefore, the mechanism of vacancy-induced H segregation serves as a helpful analogy to elucidate delayed fracture in Fe, where the sequence of vacancy diffusion to GB corresponds to the observed delayed fracture in steel in H-rich environments.

The diffusion time $\left(t_{H}\right)$ of H segregation into GB (or FS) to reach the given enrichment factor (α) was estimated based on McLean’s kinetic theory as follows [49]:

(9) $$t_{H}=\frac{9\alpha^{2}d^{2}}{16FD_{H}}$$

where α is the ratio of H content at the GB/FS site to that at the diffusion source (i.e. $\alpha =\Gamma_{\mathrm{GB}/\mathrm{FS}}\;/\;2\Gamma_{B},$ where $\Gamma_{\mathrm{GB}/\mathrm{FS}}$ represents the hydrogen concentration at GB or FS, and $\Gamma_{B}$ is the lattice hydrogen concentration. $\Gamma_{\mathrm{GB}/\mathrm{FS}}=n_{\mathrm{GB}/\mathrm{FS}}\;/\;N_{\mathrm{GB}/\mathrm{FS}}$, where $n_{\mathrm{GB}/\mathrm{FS}}$ is the number of occupied sites at the GB or FS, and $N_{\mathrm{GB}/\mathrm{FS}}$ is the number of available sites at the GB or FS. It is important to note that the definition of $N_{\mathrm{GB}/\mathrm{FS}}$ depends on the thickness of the GB or FS to be considered. The choice of this thickness directly affects the value of $N_{\mathrm{GB}/\mathrm{FS}}$, and hence the value of α), and $d\left(\approx 0.5\;\mathrm{nm}\right)$ is the thickness of the segregation region (i.e. in the case of $\sum 3\left(111\right)$, it is $n\times d_{\left(111\right)}$, where n is an integer {1,2,3.} and $d_{\left(111\right)}$ is the interplanar distance). In ∑3, the cohesive energy reduction tendency is the same as for other GBs, except for the fact that $C_{H}$ becomes higher for other GBs in the absence of vacancy. F is the configuration factor (i.e. F = 1 at FS and F=4 at GB), and $D_{H}$ is H diffusivity ($m^{2}/s$) in bcc iron estimated from the empirical equation suggested by Hirth [50]:

(10) $$D_{H}=2\times 10^{-7}\exp\left(-\frac{828}{T}\right)$$

By analogy, the time of vacancy segregation $\left(t_{V}\right)$ into GB was estimated using Eq (9), where α is the ratio of vacancy content at GB/FS site to that in the lattice (i.e. $\alpha =C_{V}^{GB/FS}\;/\;C_{V_{o}}$, where $C_{V}^{GB/FS}$ is vacancy concentration at GB/FS and $C_{V_{o}}$ is the lattice vacancy concentration. $C_{V}^{GB/FS}=n_{V}\;/\;N_{\mathrm{Fe}}$, where $n_{V}$ is the number of vacancies at the GB/FS, and $N_{\mathrm{Fe}}$ is the number of Fe atoms at the GB/FS. Similarly, the choice of GB thickness will influence the value of α), and $D_{V}$ is vacancy diffusivity ($m^{2}/s$) in bcc Fe estimated using Eq (11) [54]:

(11) $$D_{V}=\frac{4a^{2}}{3}v\exp\left(-\frac{E}{k_{B}T}\right)$$

where a is the jump length (equal to the first-neighbor atomic distance), v is the attempt frequency (equal to atomic vibrational frequency), and E is the activation barrier.

Hydrogen and vacancy diffusion were estimated using Eq (9) and (11) at 300 K, yielding $D_{H}=1.27\times 10^{-8}$ $m^{2}/s$ and $D_{V}=1.4\times 10^{-18}m^{2}/s$ (for E=0.62 eV). Accordingly, $t_{H}$ and $t_{V}$ for a GB were estimated as 0.65 s and 9.92$\times 10^{5}$ s, respectively (i.e. for calculating $t_{H}$, $n_{\mathrm{GB}}$ is 87 and $N_{\mathrm{GB}}$ is 673. Similarly, for $t_{V}$, $n_{V}$ is 19 and $N_{\mathrm{Fe}}$ is 271, for a GB thickness of 1 nm). Therefore, it is reasonable to assume that $t_{V+H}$>>$t_{H}$. Here, $t_{V+H}$, representing the time for vacancy-induced H segregation, is approximately in the same order as $t_{V}$, considering that $t_{V}$>>$t_{H}$. We used $\Gamma_{B}$ =$\;8.487\times 10^{-8}$, lattice vacancy concentration, and $C_{\mathrm{Vo}}=10^{-6}$ for the evaluation. However, accurate estimation was challenging because H and vacancies can trap each other, implying much more complex diffusivity. Under mechanical loading in the presence of H, the promotion of vacancy migration by PDLs can be a meaningful mechanism because of the higher diffusivity of PDLs (i.e. $D_{\mathrm{PDL}}\approx 3.43\times 10^{-7}$ $m^{2}/s$ for a PDL with 37 vacancies) compared to mono vacancies [25]. Accordingly, this mechanism can lead to high vacancy concentration near GBs.

**Figure 9.**
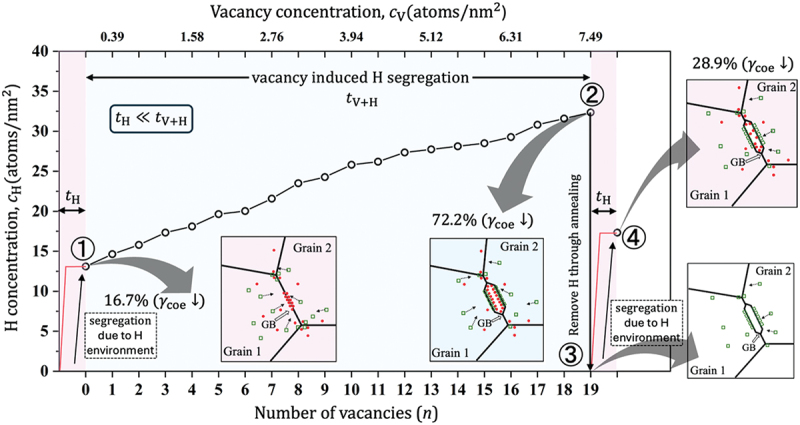
The significance of vacancy-induced H segregation to cause the delayed fracture in steel (color).

Figure 9 illustrates the proposed mechanism of delayed fracture in steel. Based on the vacancy-induced H-segregation mechanism, a quantitative comparison was used to elucidate the delayed fracture process for ∑3. When the material is exposed to an H-rich environment, H tends to segregate first ($t_{H}$<<$t_{V+H}$); however, this results in the cohesive energy reduction by only 16.7% (Figure 9, ①). Similarly, vacancies formed in the material by various dislocation activities can gradually diffuse toward the GB and trap more H atoms, as illustrated in the insets ① and ②, where the order of vacancy diffusion time is the same as the reasonable time of delayed fracture. Vacancy-induced H-segregation results in a decrease in cohesive energy of 72.2% at $C_{V}=7.491/nm^{2}$ (Figure 9, ②). Therefore, it is suitable to conclude that the vacancy-induced H-segregation mechanism can explain the delayed fracture in steel. Furthermore, the recovery of iron after vacancy-induced H-segregation can be explained using the same mechanism. We performed thermal annealing at 600 K for about 2 ns (process ② to ③ by removing the H. Then, we performed H segregation as explained in Section 2.2 (process ③ to ④). The resulting decrease in cohesive energy is only 28.9% (Figure 9, ④) owing to the removal of excess volume through annealing. Conversely, the introduction of vacancies before H results in a negligible reduction in cohesive energy (3.42%).

#### 3.2.2. Vacancy-induced hydrogen segregation and decohesion of GBs under 2% strain

Under 2% tensile strain, the optimal $C_{H}$ remains relatively consistent across GBs, yet $C_{H}$ exhibits different values than under zero strain. Specifically, a considerable increase in $C_{H}$ is observed at ∑17, whereas it slightly decreases at ∑3 and ∑9, contingent upon vacancy distribution (Figure 8(a)). As shown in Figure 7(e), at zero strain, the vacancy trap energy is relatively decreasing at different vacancy concentrations and fluctuates around the average. However, it increases with vacancy concentration at 2% tensile strain. Therefore, we concluded that vacancies are strongly bound at GBs under tensile loading. The sensitivity to vacancy-induced H segregation is prevalent across all GBs, demonstrating increased sensitivity to GB decohesion compared to that at zero strain. However, the correlation between $C_{H}$ and reduction in cohesive energy is small in almost all GBs compared to the scenario without vacancies. The reduction in cohesive energy for vacancy-induced H segregation increased by (5–28)% under strain compared to zero strain (Figure 8(b)). Therefore, the effect of tensile strain (or stress) is significant in the presence of vacancy traps, particularly, at GBs with larger misorientation angles. Under 2% strain, H segregation at ∑3 remains relatively weak; however, the decrease in decohesion (14.7%) is significant (Figure 8(b)). Therefore, decohesion depends not only on the H concentration but also on vacancy distribution (i.e. tendency to create nanovoids) at a GB under tensile strain.

**Figure 10.**
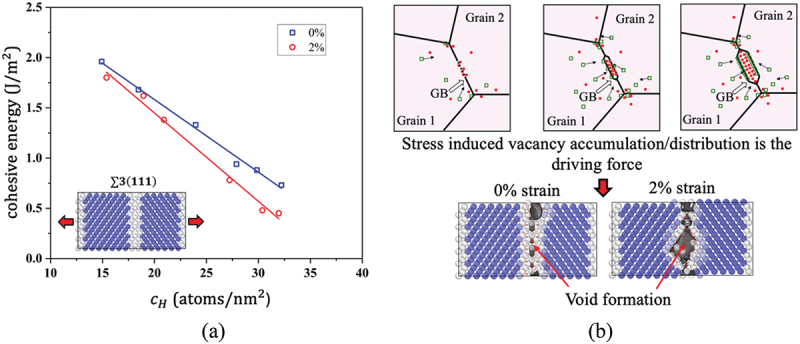
(a) Reduction of cohesive energy with H concentration at $\sum 3\left(111\right)$ and (b) influence of strain on the void formation at GB (the top three figures depict schematic representations of vacancy-induced hydrogen segregation at GB) (color).

As shown in Figure 10(a), cohesive energy decreases linearly with vacancy-induced H concentration. The reduction rate is higher under external tensile strain, suggesting a substantial effect of tensile strain on embrittlement. Precisely, the decrease in cohesive energy under the tensile strain is controlled by $C_{H}$ and stress-induced void formation at GBs. As indicated in Figure 10(b), vacancies localize and form voids under tensile strain. This reduction in the bonded GB area substantially affects decohesion, even at lower $C_{H}.$ Accordingly, the vacancy-induced H-enhanced embrittlement mechanism proposed here can explain the delayed fracture and the sensitivity of HE in steel to mechanical loading. Moreover, a recent first-principles study on Σ3 GB revealed that the tendency for a decrease in delayed fracture resistance is not just due to H and carbon atoms but is also significantly influenced by the presence of vacancies [41]. Furthermore, a 15–35% reduction in cohesive energy might be sufficient to cause fracture at very high stress levels, particularly in high-strength steels, which can trigger such failures. However, it is important to analyze the intricate interplay between solutes, H, and vacancies to understand how fractures can occur within the elastic range. This aspect is left for future studies.

Further, the relationship between the applied strain and the excess volume at the GB is influenced by the supercell size. Stress relaxation depends on the GB thickness relative to the supercell size in the normal direction to the GB and the type of GB. Employing a sufficiently large supercell or adopting a stress-controlled condition would enable more qualitative comparisons, but such methodologies are computationally expensive and beyond the scope of this study. The insights derived from our methodology still provide valuable contributions to understanding the role of H and vacancies in embrittlement phenomena. By applying a consistent strain across models, we focus on elucidating the interplay of H segregation and vacancy-mediated mechanisms in influencing the cohesive properties and failure processes at GBs.

### 3.3. Effect of mobile hydrogen on GB decohesion

**Figure 11.**
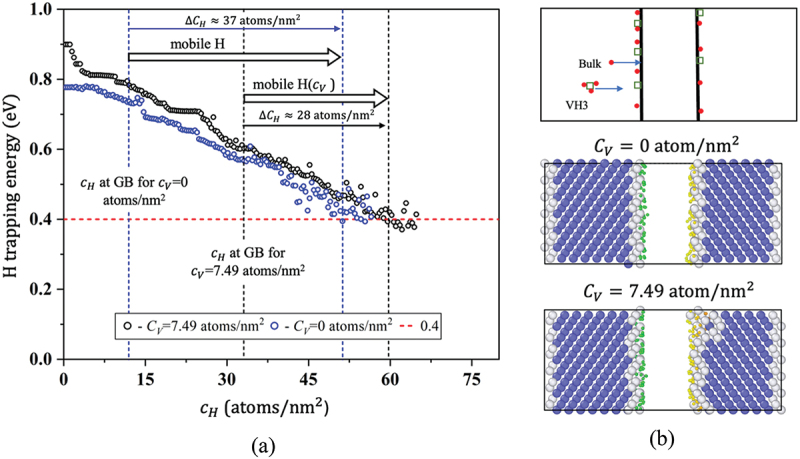
(a) Trend of H trapping energy at FS with and without vacancies (b) schematic representation of H diffusion toward FS with and without the impact of trapped vacancy (top), and saturated segregated H at FS without vacancies (middle) and with vacancies (bottom) (H atom color by position along x direction). (color).

As mentioned earlier, we consider the effect of mobile H corresponding to H diffusion from bulk to FS in ∑3. Figure 11 illustrates H trap energy at FSs comprising the GB with $C_{V}$ of 0 and $7.49\;1/nm^{2}$. Considering the mobile H atoms that diffuse from the bulk to FS, we obtained increases in $C_{H}$ of 37 and 28 $\mathrm{atom}/nm^{2}$ for the cases with and without vacancies, respectively. Here, we defined the optimal $C_{H}$ considering the total number of sites with $E_{FS,H\left(x\right)}^{\mathrm{Trap}}$ >0.4 eV. Assuming a slow fracture (i.e. mobile H), the reduction in cohesive energy was estimated using Eq (8). Estimated $\gamma_{coe}^{m,n}$ or $\gamma_{coe}^{m}$ strongly depend on H concentration both at the GB and in the bulk. We can estimate the reduction in cohesive energy and H diffusion time for a range of concentrations, but here we consider a particular $C_{H}$, chosen randomly, which is greater than the $C_{H}$ at GBs both with and without vacancies. Accordingly, for $C_{H}=46.71\;\;\mathrm{atoms}/nm^{2}$, $\gamma_{coe}^{m,n}$ and $\gamma_{coe}^{m}$ were estimated as 0.48 $J/m^{2}$ and 0.91 $J/m^{2},$ respectively, whereas in the case of immobile H, $\gamma_{coe}^{m,n}$ and $\gamma_{coe}^{m}$ were estimated as 0.8 $J/m^{2}$($C_{H}=32.36\;\;\mathrm{atoms}/nm^{2}$), and 2.19 $J/m^{2}$($C_{H}=12.81\;\;\mathrm{atoms}/nm^{2}$), respectively. We considered $\mu_{b}=k_{B}T\ln\left[\Gamma_{B}/\left(1-\Gamma_{B}\right)\right]=-0.42\;\;\mathrm{eV}$ (where $\Gamma_{B}$=$8.487\;\times \;10^{-8}$) in the evaluation. Therefore, the cohesive energy decreases in the case of mobile H. The H diffusion time required to reach the FS was estimated using Eq (11), yielding a value of ∼8.02 s for $\Delta \gamma_{coe}^{m}$ and ∼4.4 s for $\Delta \gamma_{coe}^{m,n}$, where $\Delta \gamma_{coe}^{m}$ and $\Delta \gamma_{coe}^{m,n}$ represent the decreases in cohesive energy caused by mobile H in FS without and with vacancies, respectively. However, to obtain a reasonable quantitative evaluation, the trapping effect of vacancies on H diffusion would have to be considered (Figure 11(b)). Nevertheless, a certain level of reduction in cohesive energy is easily attained by mobile H because of a vacancy-induced H concentration.

## 4. Conclusion

The H segregation at GBs of α-Fe under external strain with and without vacancy-induced effects was investigated herein. In particular, GB decohesion was evaluated in four cases: non-vacancy-induced and vacancy-induced H segregation under zero and 2% tensile strain. The main findings are summarized below:

1. The hydrogen concentration $C_{H}$ and cohesive energy across GBs strongly correlate with GB free volume without vacancy-induced effect, except for the ∑3 case where the GB free volume weakly correlates with $C_{H}$ owing to the localized nature of free volume at the GB plane. The GBs with low misorientation angles (i.e. ∑19 and ∑9) exhibited no sensitivity to external tensile strain, whereas other GBs were sensitive to increased $C_{H}$. However, the reduction of cohesive energy owing to tensile strain was minor for all GBs.
2. Vacancy migration to GBs functioned as a driving force of H trapping at GBs (vacancy-induced H segregation). The sensitivity to vacancy-induced effects was observed almost for all GBs, resulting in increased $C_{H}$.
3. The reduction in the cohesive energy was mainly determined by vacancy-induced H segregation. In particular, at the vacancy concentration of $C_{V}=7.491/nm^{2}$, the loss in cohesive energy was 70–80% and 73–95% for almost all GBs under zero and 2% tensile strain, respectively. At the same time, in the absence of vacancies, the cohesive energy decreased only by approximately 15–35%. Furthermore, vacancy segregation enhanced the effect of strain.
4. The impact of mechanical loading on HE was discussed in the context of the vacancy-induced H segregation mechanism. The stress-induced vacancy redistribution resulted in the formation of nanovoids at the GB plane.
5. The delayed fracture in steel was explained using the vacancy-induced H segregation mechanism. Particularly, the ∑3 case was used to explain the different stages of H trapping and its role in GB decohesion. Furthermore, recovery of GB decohesion was achieved after thermal annealing (removal of the trapped H) at 600 K, with a decrease in cohesive energy reduced from 72.2% to 28.9% under zero tensile strain.

## Figures and Tables

**Figure A1.**
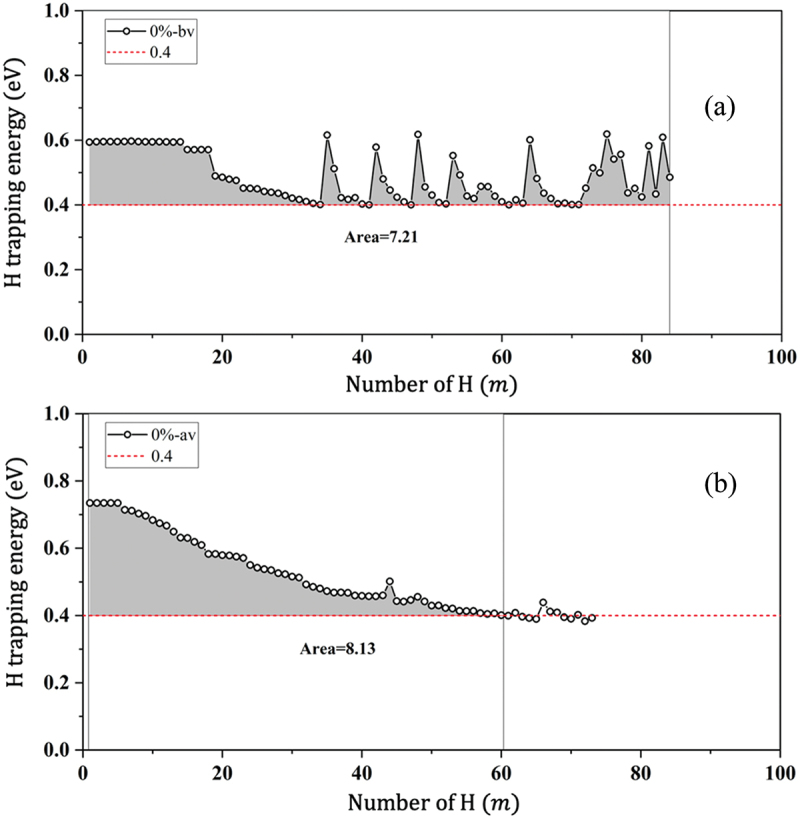
Hydrogen segregation for $\sum 3\left(111\right)$, (a) before introducing vacancy (bv), and continued to segregate after each vacancies, (b) after introducing vacancy (av), where H segregation is consider after intruding the vacancies.

**Figure A2.**
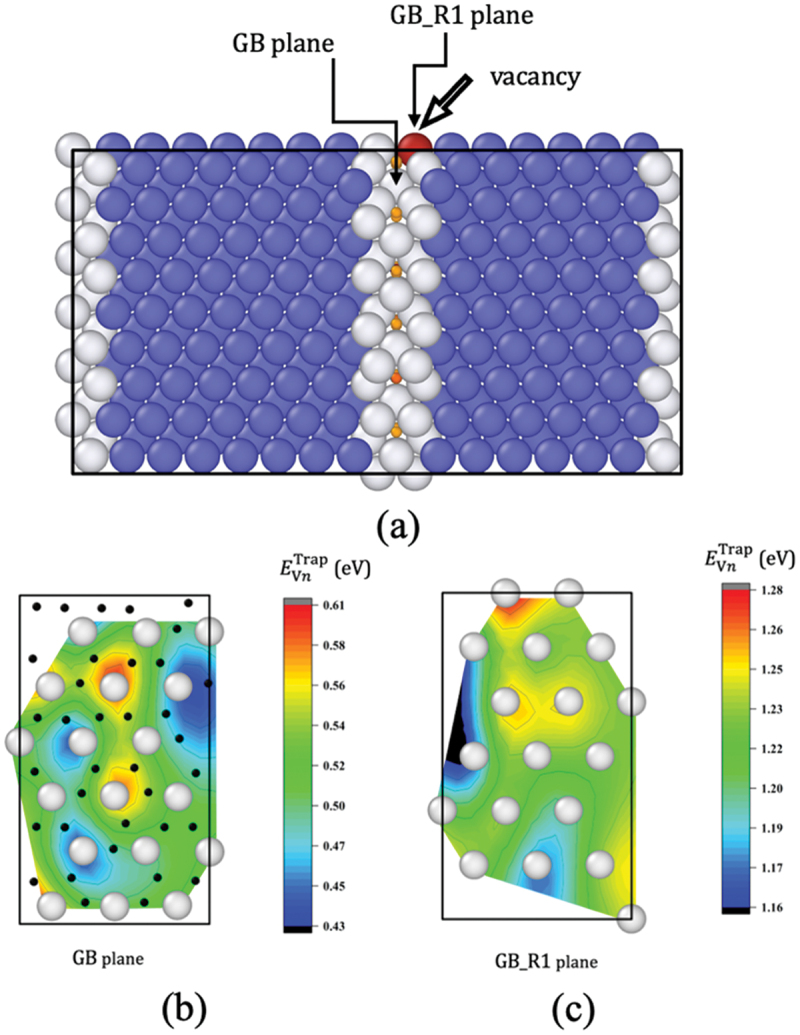
First vacancy position neat GB plane, (a) GB structure, (b) vacancy trapping energy on GB plane, and (c) vacancy trapping energy on GB_R1 plane.

**Figure A3.**
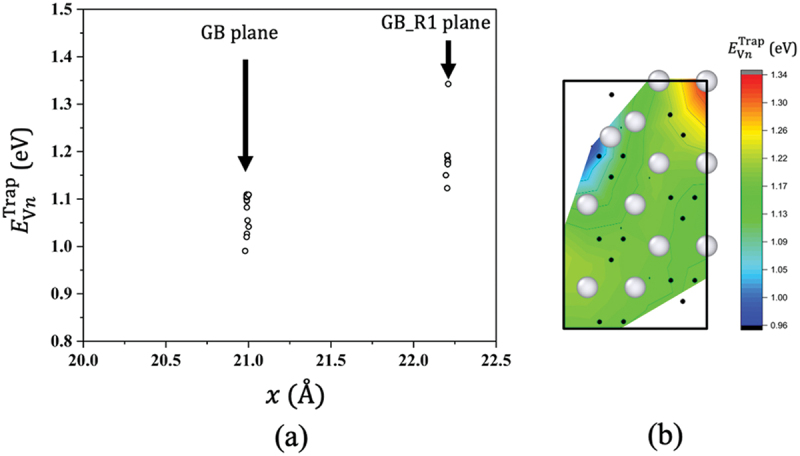
Fifteenth vacancy position near the GB plane: (a) vacancy trapping along the x direction on the GB and adjacent planes, and (b) vacancy trapping energy on the GB_R1 plane.

## Data Availability

The raw/processed data required to reproduce these findings cannot be shared at this time as they form part of an ongoing study.

## Appendix A

When the number of vacancies increases, it can alter the GB structure, where already trapped H may de-trap and trap at other stable sites. In simple terms, its distribution can vary slightly. However, in this study, we consider the GB weakening as a result of vacancy-induced H segregation, which is related to the potential energy gain by H trapping (area under Figures 5 and 7). As indicated in the Figure A1, we removed the H after introducing the vacancy and performed a similar analysis. The results show that $E_{\mathrm{gain}}$(bv)≈ $E_{\mathrm{gain}}$(av) (see Figure A1, $E_{\mathrm{gain}}$ is approximated to area under the plot), indicating that our discussion is meaningful. Additionally, we can evaluate the redistribution of H for every vacancy or after several iterations by removing some H and checking the trap site and energy of the next suitable H. Nevertheless, this process will lead to very high computational costs, and our proposed conclusions do not strongly depend on this behavior.

Further, the entropy of defects should also be included for the determination of equilibrium states of configuration or distribution of defects in materials at a finite temperature. This includes both the configurational entropy of defects and the vibrational entropy of atoms around defects. The vibrational entropy is basically neglected in most methods except for MD and we believe the effect is not significant for point defects. The former is not directly taken into account in our method, but it is justified in the case of distribution around defects at 300 K. The H occupancy at a trap site steeply increases when its trap energy increases from 0.35 eV to 0.45 eV at 70MPa and 300K. Almost all sites with a trap energy of >0.45 eV are occupied by H. On the other hand, the hydrogen occupancy is quite low at sites with <0.35 eV. As shown in Figures 5 and 7, the number of trap sites with 0.35 eV to 0.45 eV is limited. Furthermore, some hydrogen at higher trap energy (>0.4 eV) can be de-trapped, possibly trapped with trap energy less than 0.4 eV. However, we believe that the contribution to potential energy, which is used to calculate cohesive energy, approximately cancels each other. For vacancy, trap energy 1 eV, which is employed here gives, the occupancy ~1. It means vacancies are definitely trapped when they diffuse to GB. We did not consider the thermal equilibrium concentration of vacancy. Vacancy trap at GB is a time-controlled phenomenon. We assumed the trapped vacancy migrates to strongest trap sites in GB because when it makes a cluster in GB, the trap energy at the strongest site is considerably higher than distributed state (see Fig. A.2 and A.3).
